# Genome-Wide Analysis of nsLTP Gene Family and Identification of *SiLTPs* Contributing to High Oil Accumulation in Sesame (*Sesamum indicum* L.)

**DOI:** 10.3390/ijms22105291

**Published:** 2021-05-18

**Authors:** Shengnan Song, Jun You, Lisong Shi, Chen Sheng, Wangyi Zhou, Senouwa Segla Koffi Dossou, Komivi Dossa, Linhai Wang, Xiurong Zhang

**Affiliations:** 1 Key Laboratory of Biology and Genetic Improvement of Oil Crops, Ministry of Agriculture, Oil Crops Research Institute, Chinese Academy of Agricultural Sciences, Wuhan 430062, China; songsn1988@gmail.com (S.S.); junyou@caas.cn (J.Y.); shilisongning@gmail.com (L.S.); shengchen2424@gmail.com (C.S.); zhouwangyi11@gmail.com (W.Z.); dossouf@yahoo.fr (S.S.K.D.); dossakomivi@gmail.com (K.D.); 2 Shijiazhuang Academy of Agricultural and Forestry Sciences, Shijiazhuang 050041, China; 3 Laboratory of Genetics, Horticulture and Seed Sciences, Faculty of Agronomic Sciences, University of Abomey-Calavi, Cotonou 01 BP 526, Benin

**Keywords:** *Sesamum indicum*, lipid transfer proteins, transcriptomic analysis, oil content, overexpression, candidate genes

## Abstract

The biosynthesis and storage of lipids in oil crop seeds involve many gene families, such as nonspecific lipid-transfer proteins (nsLTPs). nsLTPs are cysteine-rich small basic proteins essential for plant development and survival. However, in sesame, information related to nsLTPs was limited. Thus, the objectives of this study were to identify the *Sesamum indicum* nsLTPs (*SiLTPs*) and reveal their potential role in oil accumulation in sesame seeds. Genome-wide analysis revealed 52 *SiLTPs*, nonrandomly distributed on 10 chromosomes in the sesame variety Zhongzhi 13. Following recent classification methods, the *SiLTPs* were divided into nine types, among which types I and XI were the dominants. We found that the *SiLTPs* could interact with several transcription factors, including APETALA2 (AP2), DNA binding with one finger (Dof), etc. Transcriptome analysis showed a tissue-specific expression of some *SiLTP* genes. By integrating the *SiLTPs* expression profiles and the weighted gene co-expression network analysis (WGCNA) results of two contrasting oil content sesame varieties, we identified *SiLTPI.23* and *SiLTPI.28* as the candidate genes for high oil content in sesame seeds. The presumed functions of the candidate gene were validated through overexpression of *SiLTPI.23* in *Arabidopsis thaliana*. These findings expand our knowledge on nsLTPs in sesame and provide resources for functional studies and genetic improvement of oil content in sesame seeds.

## 1. Introduction

Sesame seed oil is one of the most appreciated oils worldwide due to its quality values and the physiological functions of its specific lignans [1]. Accordingly, the demand for high oil content sesame seed is increasing considerably. To meet the demand, sesame breeders have conducted various studies at the genomics level to discover the genetic basis of sesame oil content and lay the foundation for further genetic improvements [2,3,4]. Whole-genome sequencing and assembly have provided the 274 Mb reference genome of diploid sesame, a potential “model plant” for studying traits related to oil biosynthesis in plants [2]. Genome-wide association study (GWAS) and transcriptome analysis revealed that many classes of genes, including nonspecific lipid transfer proteins (nsLTPs), are involved in oil biosynthesis, regulation and accumulation in sesame seeds [2,3].

Plant nsLTPs represent small basic secreted proteins, possessing the lipid-binding ability and participating in lipid shifting [5,6]. They are often appointed nonspecific LTPs (nsLTPs) due to their faculty to bind and transfer various phospholipids, acyl groups and fatty acids between biological membranes [7,8,9]. nsLTPs were first reported in vitro in 1975 [10]. Since then, the nsLTPs family has expanded to all land plants as one of the largest protein families, representing up to 4% of total soluble proteins [6]. nsLTPs are members of the prolamin superfamily with a molecular weight usually less than 12 kDa. They are characterized by four or five α-helices and are stabilized by four conserved disulfide bridges [8,11,12]. The α-helices are constituted of an eight-cysteine motif (ECM) backbone (C–Xn–C–Xn–CC–Xn–CXC–Xn–C–Xn–C). Generally, they are biosynthesized with an N-terminal signal peptide (21–27 amino acids) that localizes the protein to the plasma membrane exterior spaces [8,13,14]. Regarding the classification, plant nsLTPs are initially divided into two subfamilies (LTP1 and LTP2) based on the molecular size of the mature protein [8,11,12]. Subsequently, a new classification system based on the amino acid sequence similarity and cysteine residue spacing in ECM divides the nsLTPs into 11 types (type I-XI) [7,15,16].

The nsLTP genes have been widely studied in many plant species, including rice, cabbage, wheat, cotton, tomato and barley [7,16,17,18,19,20,21]. This family has many genes, generally more than 50 genes in dicots, and showed variable expressions in different developmental stages and various tissues, including seeds, leaves, flower, root and bud, as well as microspores and zygotic embryos [12,20]. Their expression patterns in the different tissues indicated their importance for plant development and reproduction [8]. However, the in vivo functions of nsLTPs are still not well established, although it has been proven their involvement in lipid metabolism [22,23], seed and pollen tube development [24,25], cutin synthesis [26], plant defense signaling [27,28,29], regulation of abiotic stresses [30], and symbiotic interactions [5]. Zachowski et al. reported that nsLTP proteins could bind and transfer fatty acids and fatty acyl-CoA, lyso derivatives, and homologous fatty acid [31]. Wang et al. reported that nsLTPs genes have been favorably selected during domestication and contributed to enhancing oil content in sesame seeds up to approximately 60% of dry weight [2]. However, the diversity and biological functions of nsLTPs in sesame, especially their role in high oil accumulation in the seed, are still not understood.

In this study, a genome-wide analysis of the nsLTP gene family in sesame was performed using sequenced data from five sesame varieties, Zhongzhi 13, Baizhima, Mishuozhima, Swetha and Yuzhi11. Comparison with the homologous nsLTP genes in *Arabidopsis thaliana* [7], *Oryza sativa* [7] and *Brassica rapa* [16] helped to characterize the sesame nsLTPs. Finally, the expression patterns of nsLTP genes were examined in different tissues, and key nsLTP genes contributing to the high oil content in sesame seeds were disclosed. The candidate gene was further validated by genetic transformation (overexpression) of *Arabidopsis thaliana*. The results of this study provided resources to insight into the biological functions of nsLTP genes in sesame, especially their roles in oil biosynthesis and accumulation in developing sesame seeds.

## 2. Results

### 2.1. Diversity, Classification and Distribution of Sesamum indicum LTPs (SiLTPs)

**Figure 1:**
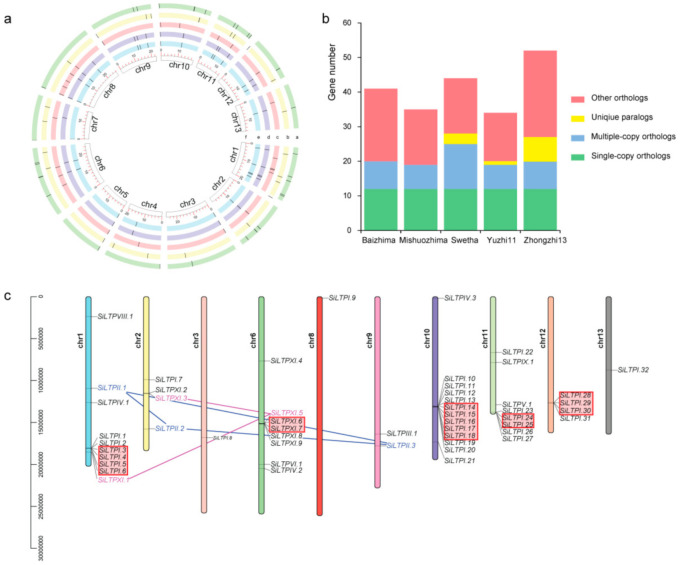
Chromosome location, ortholog and duplication analysis of sesame varieties. (**a**) From the outer edge inward, each circle represents Zhongzhi13, Swetha, yuzhi11, Baizhima, Mishuozhima and the 13 chromosomes. The black lines in each circle represent nsLTP genes. The red scale bar in the innermost circle represents 20 Mb nucleotides. The chromosome number is next to each chromosome. (**b**) Ortholog analysis of five sesame varieties. The different color of the histogram represents different types of orthologs. (**c**) Chromosome location and duplication analysis of 52 *SiLTPs* in sesame variety Zhongzhi13. The location of each *SiLTP* is indicated by a black horizontal line. The tandem duplications were represented by red boxes. The segmental duplications were represented by blue and rose red lines. The blue and rose red represent a set of segmental duplication events, respectively.

To reveal the sesame nsLTPs, we downloaded the whole protein sequence data of five sesame varieties (Zhongzhi 13, Baizhima, Mishuozhima, Swetha and Yuzhi11) from Sinbase2.0–Sesame Multi-Omics Database (http://www.sesame-bioinfo.org/Sinbase2.0/, accessed on 24 July 2019). The *SiLTPs* were specifically identified by combining the hidden Markov model (HMM) and Protein Basic Local Alignment Search Tool (BLASTP) analysis. There were 52, 42, 35, 44 and 34 nsLTP genes in Zhongzhi 13, Baizhima, Mishuozhima, Swetha and Yuzhi11, respectively (Figure 1a, Table S1). The homology analysis revealed the five sesame varieties shared 12 single-copy orthologs (Figure 1b). The varieties Swetha, Yuzhi11 and Zhongzhi13 contained three, one and seven unique paralogs, respectively and were distinguishable from Baizhima and Mishuozhima (Figure 1b).

Considering the relative high-quality assembly of the Zhongzhi 13 genome, its 52 nsLTPs were selected for further analysis. Based on the amino acid sequence similarity and cysteine residue spacing in ECM, the 52 *SiLTPs* in “Zhongzhi 13” were classified into nine subfamilies [7]. Type I, II, IV and XI constituted 32, 3, 3, and 9 genes, respectively, while the other five types (types III, V, VI, VIII and IX) constituted only one gene each. We then named the 52 *SiLTPs* based on the classification and their distribution on the chromosomes as *SiLTPI.1*, *SiLTPII.1* (Table S1). The *SiLTPs* genes were distributed nonrandomly on the 13 chromosomes (Figure 1a,c). 75.0% of the *SiLTPs* located on four chromosomes Chr1, Chr6, Chr11 and Chr13. No *SiLTP* was located on Chr 4, Ch5, and Chr7. Gene duplication plays an important role during gene evolution in plants [32,33]. Therefore, we checked the tandem duplication and segmental duplication events. Among the 52 *SiLTPs* genes, 16 genes exhibited tandem duplication on chromosomes 1, 6, 10, 11 and 12 (Figure 1c) at the locations where gene aggregation occurred. It suggested that the gene cluster may be caused by tandem duplication. Segmental repeat events were observed between different sesame chromosomes and occurred in type II and XI subfamilies on three different chromosomes (Figure 1c). The results indicate that some *SiLTPs* were produced by gene duplication events, which were the main driving forces in the evolutionary history of the *SiLTP* family.

### 2.2. Characterization and Phylogenetic Analysis of the Conserved Motifs of Sesame SiLTP Genes

**Figure 2:**
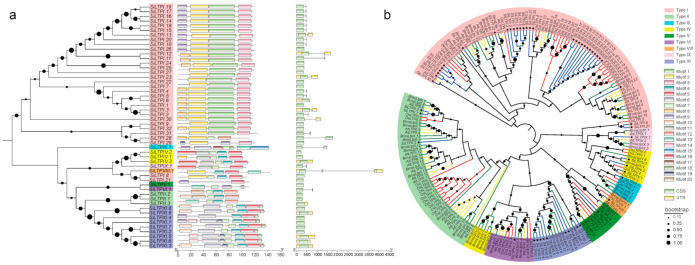
Phylogenetic relationships, gene structure and motif compositions of *SiLTPs*. (**a**) Left: phylogenetic tree of 52 *SiLTPs*. Different color of arcs represents different types of *SiLTPs*. Black dot represents the clades support values in the phylogenetic trees. Middle: conserved motif composition of SiLTPs. Different colors of boxes represent different motifs. Gray lines represent the nonconserved sequences. Scale bar at the bottom represents 20aa. Right: intron–exon structure of *SiLTPs*. Green boxes represent exon, gray lines represent introns, and yellow boxes represent UTR. Scale bar at the bottom represents 500 bp. (**b**) Phylogenetic tree of *A. thaliana*, *B. rapa*, *O. sativa* and 52 *SiLTPs* amino acid sequences. Different color of arcs represents different types of nsLTPs. Different color of clades represents different varieties. Stars represent the genes of the sesame variety Zhongzhi13. Black dot represents the clades support values in the phylogenetic trees.

To distinguish the *SiLTP* genes, we examined their conserved motifs. In general, all the genes conserved the cysteine spacing patterns (Table S1). We then evaluated the ECM structure by investigating the amino acid length, signal peptide (SP) length, mature protein (MP) length, molecular weight (MW), theoretical isoelectric point (PI) of the *SiLTPs*. We observed a variation of all the evaluated parameters (Table S1). For instance, the amino acid length ranged from 94 to 145 amino acids (aa), averaged 119 aa. The SP and MP lengths ranged from 19 to 29 aa and 68 to 120 aa, respectively. The MW varied from 9.92 to 16.33 kDa, and the PI varied from 3.83 to 10.45. To specifically differentiate the *SiLTP* genes, we selected and examined 20 hypothetical motifs of each gene using the MEME web server. We observed that some types of *SiLTPs* shared similar conserved motif organization (Figure 2a). The type I *SiLTPs* genes shared motif 2, while those classified type XI shared motif 5, 14 and 15. The number of exons in *SiLTPs* genes varied from 1 to 3 (Figure 2a). All members of types II and XI contained one exon. Members of types I, IV, V, VI contained 1–2 exon(s). All members of types III and VIII contained two and three exons, respectively. In general, genes belonging to the same type had similar gene structures and conservative motif compositions, which supported the classification of these genes.

Based on the amino acid sequences of ECM domains, the phylogenetic tree was constructed to investigate the phylogenetic relationship among the *SiLTPs* using the neighbor-joining (NJ) method (Figure 2a). Different types of *SiLTPs* were obviously separated into different groups. Except for type I, the other types formed monophyletic groups. By including the nsLTP genes from *A. thaliana* [7], *B. rapa* [16], and *O. sativa* [7] (Table S2), the new NJ tree of different species also showed similar groups, and different types of *SiLTPs* clustered together (Figure 2b). We also studied the classification and phylogenetic relationship of nsLTP proteins in the other four sesame varieties. The classification and phylogenetic tree structure of nsLTP proteins were basically consistent with that of Zhongzhi13 (Figure S1, Table S1).

### 2.3. Cis-Acting Elements and Interacting Transcription Factors among the SiLTPs

**Figure 3:**
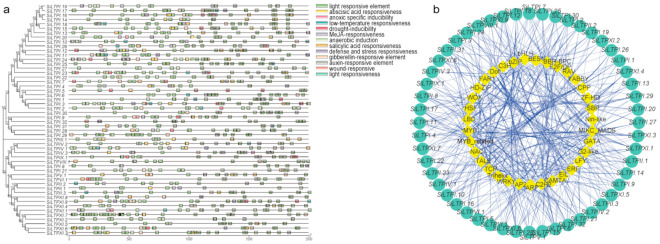
Prediction of analysis of *cis*-acting elements and prediction of transcription factors among the *SiLTPs*. (**a**) The *cis*-acting elements detected in the promoter region of each *SiLTP*. (**b**) Regulation networks between *SiLTP* and potential transcription factors. Gene IDs in the green circle refer to *SiLTPs*, and the genes in the yellow diamond represent the combining transcription factors. Relationships between *SiLTPs* and transcription factors were represented by blue lines.

To study the regulation and expression characteristics of *SiLTPs*, the *cis*-acting elements were predicted in the upstream 2000 bp of the promoter region. In total, 46 *cis*-acting elements were predicted (Figure 3a). These *cis*-acting elements were mainly related to light response, hormone response, environmental response, development, and other functions, suggesting that *SiLTP* genes may have multiple biological functions.

As *cis*-acting elements can effectively improve the accuracy and efficiency of gene transcription in combining with transcription factors, we thus predicted the transcription factors that may be involved in regulating *SiLTP* genes using the online tool PlantRegMAP (Figure 3b). Most *SiLTPs* could interact with multiple transcription factors, suggesting that they may be involved in many physiological processes. Among the predicted transcription factors, AP2, basic Helix-Loop-Helix (bHLH), C2H2 zin finger (C2H2), Dof, MIKC-type MADS-box (MIKC_MADS), v-myb avian myeloblastosis viral oncogene homolog (MYB) and NAM, ATAF1,2, CUC (NAC) were the most abundant. AP2 and Dof can increase the total lipids content in seeds [34,35,36,37,38,39].

### 2.4. Expression Profiles of SiLTP Genes in Different Tissues of Sesame

**Figure 4:**
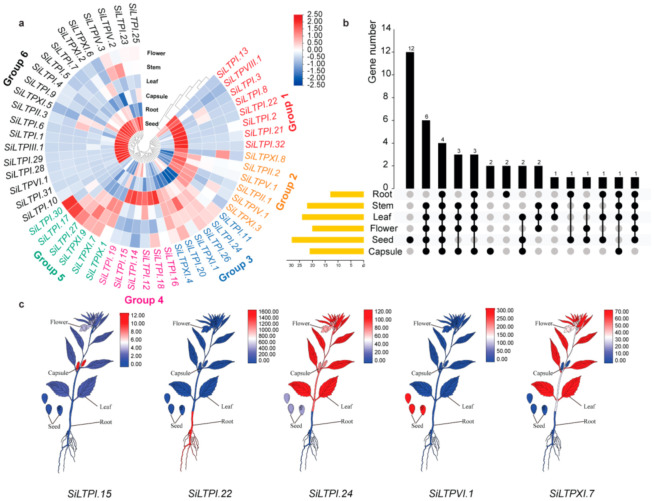
Expression patterns of *SiLTP* genes in different tissues in sesame. (**a**) Heatmap representation and hierarchical clustering of *SiLTPs* in different tissues. Different color of *SiLTP* gene IDs represents different expression patterns. Color scale represents the relative signal intensity of log10-transformed RPKM values. (**b**) Number of *SiLTP* genes that were expressed in each tissue. Yellow bar on the left indicates the whole expressed gene number in each tissue. Black dot means the expressed genes in this tissue and the black bar in the top mean the number expressed in different tissue group. (**c**) Randomly selected 5 *SiLTPs* to show the expression pattern. Color scale represents the relative signal intensity of RPKM values.

To determine the in vivo potential function location of the 52 *SiLTPs*, we examined the expression profiles in six different tissues (root, stem, leaf, flower, seed and capsule) of “Zhongzhi 13”. The genes with RPKM (Reads Per Kilobase per Million) value ≥5 in one tissue were used to ensure the reliability of the gene expression pattern. As shown in Figure 4a, a total of 42 *SiLTPs* genes were expressed in the six tissues. Notably, 13, 22, 24, 20, 28 and 21 *SiLTPs* were expressed in sesame root, stem, leaf, flower, seed and capsule, respectively (Figure 4b). Some of the *SiLTP* genes are tissue-specific (Figure 4b,c). For example, *SiLTPI.22* was only expressed in roots, while *SiLTPVI.1* was only expressed in seeds. In general, *SiLTPs* genes could be divided into six groups (groups 1–6) according to their expression patterns (Figure 4a). Group 1 consisted of eight genes, six of which were highly expressed in root, while *SiLTPI.3* and *SiLTPI.22* were only expressed in the root (Figure 4c). Group 2 (6 genes) constituted of *SiLTPs* genes expressed highly in root and capsule, such as *SiLTPXI.3*. Group 4 included four genes that exhibited a specific high expression in capsules and two genes (*SiLTPI.14* and *SiLTPI.18*) expressed in capsules only. Group 3 (6 genes) and 5 (6 genes) were composed of genes that showed high expression in more than three tissues, such as *SiLTPXI.7* and *SiLTPI.24* (Figure 4c). The genes in group 5 were only expressed in flowers, stems and leaves. There are a total of 20 genes in group 6 (Figure 4a). Based on Figure 4b, twelve genes were specifically expressed in seeds, such as *SiLTPVI.1* (Figure 4a–c). In general, more than half of *SiLTP* genes, including all the genes of group 6, and half of the genes in groups 3 and 4 were highly expressed in seeds. These results suggested that *SiLTPs* may play a role in sesame seed development.

### 2.5. The SiLTPs Genes Involved in High Oil Content in Sesame Seeds

**Figure 5:**
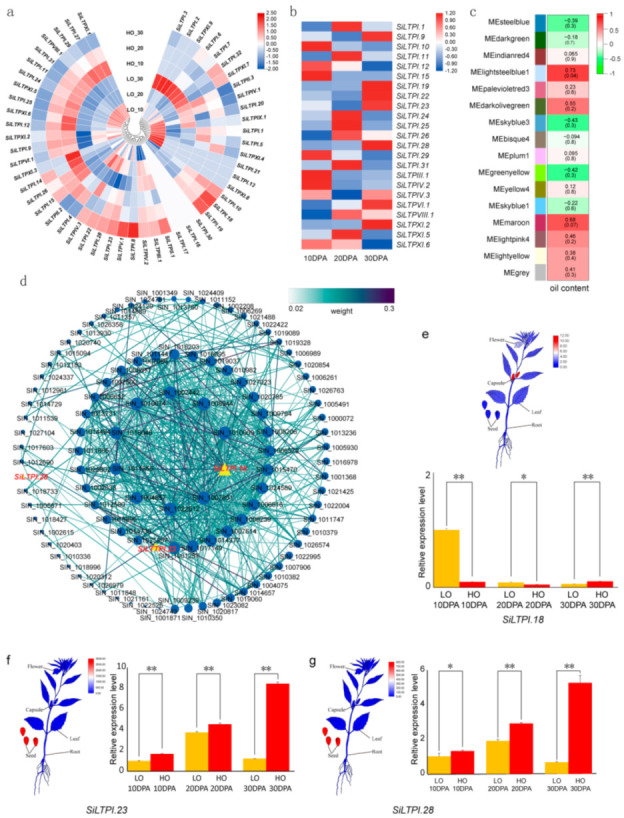
Expression patterns of *SiLTP* genes in high and low oil content sesame. (**a**) Heatmap representation and hierarchical clustering of *SiLTPs* in high and low oil content sesame seeds. The color scale represents the relative signal intensity of log10-transformed RPKM values. HO: high oil content sesame ZZM4728; LO: low oil content sesame ZZM3495. (**b**) The upregulated *SiLTPs* in at least one period of HO sesame. Heatmap representation of the log10-transformed ratio of *SiLTPs* expression in high and low oil content sesame seeds. (**c**) Heatmap of the correlation of WGCNA modules with oil content. (**d**) Cystoscope representation of co-expressed genes in the lightsteelblue1 module, which with the highest correlation with oil content (cor = 0.73). Gene IDs in yellow triangles refer to *SiLTP* genes, and the genes in the blue dot represent other sesame genes. The weight values between genes were represented by lines in different colors. (**e**) Expression levels of *SiLTPI.18*. Top: tissue expression levels of *SiLTPI.18*. Bottom: relative expression levels of *SiLTPI.18* were analyzed by qRT–PCR, using sesame histone *H3.3* gene as the internal control. Error bars represent the standard deviations of three replicates. Asterisks indicate significant expression difference between HO and LO sesame seeds (*n* = 3, *, 0.01 < *p* < 0.05; **, *p* < 0.01). (**f**) Expression levels of *SiLTPI.23*. Left: tissue expression of *SiLTPI.23*. Right: relative expression levels of *SiLTPI.23* analyzed by qRT–PCR. (**g**) The t expression levels of *SiLTPI.28*. Left: tissue expression of *SiLTPI.28*. Right: relative expression levels of *SiLTPI.28* analyzed by qRT–PCR.

To study whether *SiLTPs* are involved in the variation of sesame oil content, we examined the expression profiles of 52 *SiLTPs* in the published transcriptome data of a high oil content (HO) and low oil content (LO) sesame varieties at different seed development stages (Figure 5a) [4]. We observed that the number of expressed *SiLTP* genes in HO and LO sesame seeds both decreased from 10 days after-anthesis (DPA) to 30 DPA (Figure S2), indicating *SiLTPs* are more active during the seed development stages than the seed maturity stage. There were 26 differentially expressed *SiLTPs* genes, including eighteen *SiLTPIs*, one *SiLTPIII*, two *SiLTPIVs*, one *SiLTPVI*, one *SiLTPVIII* and three *SiLTPXIs* between the HO and LO sesames (Figure 5a). Among the differentially expressed genes (DEGs), 23 (except *SiLTPI.5*, *SiLTPI.6* and *SiLTPI.32*) were upregulated at least at one stage of HO seed development (Figure 5b). Seven of the 12 *SiLTPs* expressed specifically in seed (Figure 4a) exhibited a high expression in HO. In addition, we found that five *SiLTPs* (*SiLTPI.10*, *SiLTPI.15*, *SiLTPI.19*, *SiLTPI.26* and *SiLTPXI.2*) were upregulated at all three seed development stages in HO (Figure 5b).

WGCNA was used to analyze the coregulatory gene modules in HO and LO sesames, and a total of twelve modules were detected to have a high similarity of expression (Figure 5c). The module with the highest correlation with oil content was colored as lightsteelblue1 (Pearson’s correlation coefficient = 0.73). In this module, there were a total of 108 genes, including 3 *SiLTP* genes (*SiLTPI.18*, *SiLTPI.23*, *SiLTPI.28*). The calculated weight value and the co-expression relationships of the genes showed that *SiLTPI.18* had the highest value and *SiLTPI.28* had the smallest weight value (Figure 5d). *SiLTPI.18* showed a very low expression in seeds but a high expression in capsules (Figure 5e). *SiLTPI.23* and *SiLTPI.28* were both upregulated in HO and expressed highly in seeds (Figure 5f,g). They all belonged to group 6. In addition, they exhibited a high expression at all three stages of both HO and LO seed development (Figure 5f,g). Compared with LO, *SiLTPI.23* was upregulated at 10 and 30DPA (Figure 5f), and *SiLTPI.28* was upregulated at 30DPA in HO (Figure 5g). Considering the expression profiles and WGCNA analysis, *SiLTPI.23* and *SiLTPI.28* might be the candidate genes associated with oil content variation in sesame seeds. The qRT–PCR results of *SiLTPI.18*, *SiLTPI.23* and *SiLTPI.28* in both HO (ZZM4728) and LO (ZZM3459) sesames confirmed the reliability of the RNA-seq Data (Figure 5e–g).

**Figure 6:**
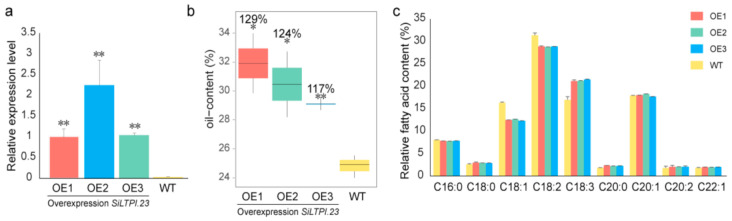
Result of *SiLTPI.23* overexpressed in *Arabidopsis*. (**a**) Relative expression levels of *SiLTPI.23 in Arabidopsis* T2 WT and *SiLTPI.23* OE lines, analyzed by qRT–PCR. Error bars represent the standard deviations of three replicates. Asterisks indicate a significant difference from the WT (*n* = 3, **, *p* < 0.01). (**b**) Seed oil content of the *Arabidopsis* T2 WT and *SiLTPI.23* OE lines. Error bars represent the standard deviations of three replicates. The value on the column represents the percentage of FA content in WT seeds. Asterisks indicate a significant difference from WT (*n* = 3, *, 0.01 < *p* < 0.05; **, *p* < 0.01). (**c**) Relative fatty acid content of the *Arabidopsis* T2 WT and *SiLTPI.23* OE lines. Error bars represent the standard deviations of three replicates.

Considering the expression pattern and higher weight value, we selected *SiLTPI.23* to verify its function in oil accumulation in sesame seed through the *Arabidopsis* transgenic experiment. We overexpressed the *SiLTPI.23* in *Arabidopsis* and selected three transgenic lines OE1, OE2 and OE3, based on *SiLTPI.23* expression patterns (Figure 6a). Compared with the seed oil content of WT (the control), the oil contents in OE1, OE2 and OE3 were significantly increased by 29, 24 and 17%, respectively (Figure 6b). There was no significant difference in fatty acid composition between OE lines and WT seeds, suggesting that the overexpression of *SiLTPI.23* did not affect the desaturation or elongation of fatty acids. However, we noticed that the content of C18:0 FA (fatty acid), C18:3 FA, C20:0 FA, C20:2 FA and C22:1 FA increased, while the content of C16:0 FA, C18:1 FA and C18:2 FA decreased in the OE lines (Figure 6c). These findings indicated the overexpression of *SiLTPI.23* in *Arabidopsis* increased the oil content in *Arabidopsis* seeds, regulated the ratio of fatty acids, and did not cause fatty acid modifications.

## 3. Discussion

Sesame (*Sesamum indicum* L.) is an important oil crop with a long history of cultivation in tropical and subtropical areas [40]. Due to its high oil content, high protein proportion, and the presence of sesamin and sesamolin, sesame is an important source of oil and has good nutritional and therapeutic effects [1,2,3,4,41]. The oil content of the dry seed is about 60% [40]. However, due to the increasing market of sesame oil, improving the sesame seeds oil content is one major objective in sesame breeding. This study provided essential information related to nsLTPs in sesame.

The plant nsLTPs were first purified from spinach leaves and named by their ability to bind and transfer a wide range of lipids across bilayers [8,10]. There were 37, 49 and 63 nsLTP genes in *A*. *thaliana*, *O*. *sativa* and *B*. *rapa*, respectively [7,16]. Here, we identified 52, 42, 35, 44 and 34 nsLTPs in *Sesamum indicum* (*SiLTPs*) varieties Zhongzhi13, Baizhima, Mishuozhima, Swetha and Yuzhi11, respectively. Based on previous classification rules [8,14,16,17,18,19,42], the 52 *SiLTPs* of Zhongzhi13 were classified into nine types (types I–VI, VIII, IX and XI), among which types I and XI were the most abundant. The classification of *SiLTPs* was also supported by the phylogenetic tree, conserved motifs and gene structures. In *A. thaliana*, *B. rapa* and *O. sativa*, types I and II accounted for the largest proportions of nsLTP genes. There were no type VII or X nsLTP in sesame. Some types were found only in certain species. To date, type VII has not been reported in dicots and was previously assumed to be unique to monocots [15]. Type X was a specific subfamily of Solanaceae [15,20].

Gene duplication is a mutation form of genome region replication, which is considered one of the main mechanisms of producing new genetic material in the process of molecular evolution [43,44]. There are many mechanisms of gene duplication, mainly including whole-genome duplication (WGD), tandem duplication, segmental duplication, transposon-mediated duplication and retroduplication. WGD is an extreme mechanism of gene duplication, which leads to the sudden increase of genome size. However, this is not the only mechanism for producing duplicate genes. In plant genomes, tandem and segmental duplication play an important role in the evolution and expansion of gene families [32,33,43,44]. Tandem duplication is mainly caused by chromosome recombination and results in a cluster of two or more homologous sequences. Members of a gene family formed by tandem duplication are usually closely arranged in the same intergenic region or adjacent intergenic region. However, the segmental duplication is produced by polyploidy or chromosome rearrangement, which leads to the duplication of genes far away, even in different chromosomes [44,45,46]. Five tandem duplication events, including 16 genes, and five segmental events involving six genes were observed in sesame. Similar tandem duplication events (7, including 17 genes) and segmental events (15 involving 24 genes) were reported In *B. rapa* [16]. This suggests that differences in tandem and segmental duplication events may be responsible for the different number of nsLTPs in sesame and *B. rapa*. The expression analysis of *SiLTPs* indicated that they might participate in developing different sesame tissues. However, we observed a tissue-specific expression of some *SiLTP* genes. The tandem duplication gene pairs *SiLTPI.4*, *SiLTPI.5* and *SiLTPI.6* on chr1 were expressed specifically in seeds, suggesting they may play critical roles during sesame seed development. All Type II genes occurred segmental duplication events. The expression patterns of *SiLTPII.1* and *SiLTPII.2* were similar, and their expression levels were higher in roots and capsules. These results suggested that genes with similar duplication events may have similar functions during the sesame plant development. It was previously noticed that nsLTP genes could be expressed in various tissues and were involved in many biological processes, including lipid metabolism, seed development, adaptation to biotic and abiotic stresses, etc. [23,24,47].

To further understand the potential biological functions of *SiLTPs*, we predicted the *cis*-acting elements and transcription factors. All the detected *cis*-acting elements were related to biological processes. The major transcription factors we predicted included AP2, bHLH, C2H2, Dof, MIKC_MADS, MYB and NAC. Among them, AP2 is the unique transcription factor family that is important for the whole life cycle of plants. Reports indicated that it is involved in developmental processes and plant responses to various stresses [34,35,36]. WRINKLED1 (WRI1), an AP2 transcription factor, was involved in carbon metabolism, fatty acid synthesis and triacylglycerols storage in the seed of oil plants [34,35,36]. Dof is a known transcription factors family regulating various biosynthetic pathways at the transcriptional level [37]. Previous studies reported that overexpression of Dof-type transcription factor genes could increase the total lipids content in seeds [37,38,39]. bHLH, MYB and NAC transcription factors that interact with the ABA signaling pathway could not only regulate the biosynthesis of many phytochemicals, including flavonoids, lignin and lignans but also help plants to adapt to abiotic and biotic stresses [48,49]. Together with the above results, we speculated that *SiLTPs* might be involved in mineral nutrition, plant growth and reproduction, adaptation to various stresses, seed oil and bioactive compounds biosynthesis and accumulation in sesame. Further experiments are needed to precisely detect their functions in each developmental process.

In crop seeds, fatty acids are synthesized in the chloroplasts and endoplasmic reticulum, and the oil is stored as triacylglycerol in the oil bodies [50,51,52]. The above indicated that some *SiLTP* genes could influence fatty acid biosynthesis and oil content in sesame seeds by interacting with various transcription factors. In rice, it was reported that reducing expressing *OsLTP36* (*Os03g25350*) led to the decrease of seed fatty acids content and the increase of chalkiness rate [23]. In sesame, previous studies reported that nsLTPs are involved in lipid transfer and oil content [2]. Choi et al. reported sesame nsLTP genes were involved in lipid transfer into the extracellular matrix [53]. Wei et al. identified two *SiLTPs* (*SiLTPI.15* and *SiLTPVI.1*) as candidate causative genes underlying oil content variation in sesame [3]. Wang et al. reported that an expansion of type I nsLTP genes was responsible for the higher oil content in sesame seeds than other oil crops [2]. In this study, through the transcriptome and WGCNA analysis of HO and LO sesame varieties, we identified *SiLTPI.23* and *SiLTPI.28* as the candidate genes associated with high oil content in sesame. The expression of *SiLTPI.23* and *SiLTPI.28* in seeds were higher than other tissues, and their expression levels in HO sesame seeds were higher than those in LO sesame seeds. The potential oil increasing function of *SiLTPI.23* was validated through overexpression in *Arabidopsis*. The overexpression of *SiLTPI.23* improved the oil content and regulated the ratio of fatty acids in transgenic *Arabidopsis* seeds. AP2 and Dof transcription factors were predicted in the *SiLTPI.23* and *SiLTPI.28* upstream 2000 bp promoter sequence. These findings suggest that *SiLTPI.23* and *SiLTPI.28* may interact with AP2 and Dof transcription factors to promote oil accumulation in sesame seeds by inducing fatty acids and other lipids biosynthesis in developing seeds and by enhancing their transfer from other sesame tissues into developing seeds. This study provided important genetic resources for further understanding the molecular mechanism of sesame oil accumulation and genetic improvement of sesame oil content.

## 4. Materials and Methods

### 4.1. Identification of the nsLTP Genes in Sesame Genome

The whole sesame protein file was downloaded from Sinbase2.0—Sesame Multi-Omics Database (http://www.sesame-bioinfo.org/Sinbase2.0/, accessed on 19 June 2019). The hidden Markov model (HMM) profiles PF14368 and PF00234 were used as hmmsearch queries (*p* < 0.001; http://hmmer.org, accessed on 7 July 2019). For hmmsearch, the default parameters were adopted, and the cutoff value was 1E−5. To avoid the possible loss of the nsLTP gene due to an incomplete ECM domain, a local BLASTP was performed with a cutoff of 1E−5, using the published *Arabidopsis thaliana*, *Oryza sativa* and *Brassica rapa* nsLTP amino acid sequences as queries [7,16]. After removing the repeated sequences, all assumed nsLTP proteins were submitted to SignalP 5.0 (http://www.cbs.dtu.dk/services/SignalP/, accessed on 24 July 2019) to confirm the presence of signal peptides. Considering the low molecular weight of nsLTP proteins, the mature proteins containing more than 120 amino acids were excluded. All candidate nsLTPs were then submitted to Pfam 32.0 (http://pfam.xfam.org, accessed on 29 July 2019) to confirm the LTP domains and to Batch Web CD-Search Tool (https://www.ncbi.nlm.nih.gov/Structure/bwrpsb/bwrpsb.cgi, accessed on 29 July 2019) to confirm the AAI_LTSS structure. Finally, each candidate *SiLTP* gene was examined manually to ensure the presence of ECM sequences and to eliminate the protein lacking in the essential ECM structure. In addition, the theoretical isoelectric point and molecular weight were calculated by using the Compute pI/Mw tool in ExPASy (http://web.expasy.org, accessed on 26 November 2019).

### 4.2. Sequence Analysis, Phylogenetic Analysis and WGCNA Analysis

The chromosome location and gene structure information of *SiLTPs* were based on the annotation downloaded from Sinbase2.0. The nsLTP genes were mapped on the chromosomes and named according to their positions. The positions of *SiLTPs* on chromosomes of the sesame genome were drawn using Circos [54] and TBtools (https://github.com/CJ-Chen/TBtools, accessed on 16 November 2019) [55]. Boutrot’s classification system was used to classify the sesame nsLTPs into subgroups [7]. We used OrthoMCL to predict orthologs and paralogs of the five sesame varieties, and the homology inference algorithm of OrthoMCL is to predict orthogroups [56]. An orthogroup is a group of genes derived from a single gene in the last common ancestor of all species. If a homologous group contains all the species studied, and each species has only one gene, it is a group of single-copy orthologs. If a homologous group contains all the species studied, and any species has more than one gene, it is a group of multiple copy orthologs. If a homologous group does not contain all the species studied, it is a group of other orthologs. Tandem duplication and segmental duplication were analyzed by multiple collinear scanning toolkits (MCScanX) [57]. First, sesame-to-sesame proteins were compared using whole-genome BLASTP, and the parameters were E-value = 1e−5 and Num_alignments = 5 [58]. Then MCScanX was used to identify the putative homologous gene pairs and collinear blocks using default parameters. Finally, TBtools was used to visualize the MCScanX results [55]. Tandem duplication and segmental duplication were distinguished by genome distribution. Tandem duplication refers to paralogous genes that are adjacent to each other on the same chromosome. Moreover, segmental duplication is defined as paralogous genes that are far away from each other at different positions on chromosomes [57].

The conserved motifs of nsLTP proteins were analyzed using MEME Suite 5.0.4 online program (Multiple Em for Motif Elicitation, http://alternate.meme-suite.org/tools/meme, accessed on 21 September 2019). The exon–intron organization of sesame nsLTPs was analyzed by Gene Structure Display Server (GSDS: http://gsds.cbi.pku.edu.cn, accessed on 25 September 2019). All nsLTP amino acids alignment was performed by Mafft v7.158b (http://mafft.cbrc.jp/alignment/software/, accessed on 13 August 2019) using the G-INS-i algorithm [59]. The conservative sequence of ECM was edited manually by MEGA-X [60]. Then, the phylogenetic relationships among the nsLTPs were constructed using the neighbor-joining method in MEGA-X software with the following parameters: Poisson model, pairwise deletion, and 1000 bootstrap replications. The phylogenetic trees were visualized using the iTOL web package (https://itol.embl.de/, accessed on 20 January 2021) [61]. The weighted correlation network analysis (WGCNA) is performed in R (version 4.0.2), and the R-packet WGCNA is used to generate the signed weighted correlation network [62]. The default power is twenty-six.

### 4.3. Plant Growth, RNA Extraction and SiLTPs Expression Analysis

To detect the expression level of *SiLTPs* in sesame with high and low oil content, we selected the same sesame varieties with transcriptome analysis, among which the high oil content (HO) sesame variety is ZZM4728 (Zhongzhi13), and the low oil content (LO) sesame variety is ZZM3459. The RNA-seq Data of the HO and LO sesames were available [2]. The seeds of the sesame varieties were provided and preserved by the sesame germplasm resources, China National Gene Bank, Oil crops Research Institute, Chinese Academy of Agricultural Sciences, Wuhan, China. The sesame plants were grown under normal growth conditions at the experimental station of the Institute in Wuhan, Hubei Province, China from May to September 2019. For HO and LO sesames, their developing seeds were sampled at the early stage (10 DPA, days after-anthesis), middle stage (20 DPA) and later stage (30 DPA), and fast-frozen in liquid nitrogen for RNA extraction. The frozen materials were ground in liquid nitrogen. Total RNA of each sample was extracted with EASYspin Plus plant RNA kit (Aidlab, Beijing, China). The HiScript II 1st strand cDNA synthesis kit (Vazyme Biotech, Nanjing, China) was used to reverse translated the RNA. Three potential candidate genes (*SiLTPI.18*, *SiLTPI.23* and *SiLTPI.28*) were selected for the qRT–PCR. The qRT–PCR was performed using ChamQ™ SYBR1 qPCR master mix (Vazyme Biotech, Nanjing, China) on LightCycler480 real-time PCR system. The sesame histone *H3.3* gene (SIN_1004293) was used as the internal control [63]. The expression level of the SiLTP genes was quantified following the 2^-ΔΔCT^ method [64]. The primers used for the qRT–PCR are shown in Table S3.

### 4.4. Arabidopsis Transgenics Experiment

To validate the potential function of *SiLTPI.23* in transgenic *Arabidopsis thaliana*, its coding sequence was isolated from Zhongzhi13 (HO) by PCR using the primers presented in Table S4. pCAMBIA 1301s vector was used for gene cloning [65]. The recombinant vector was transferred into *Agrobacterium tumefaciens* LBA4404 before *Arabidopsis thaliana* genetic transformation. The positive transgenic plants were screened on Murashige and Skoog (MS) medium containing 50 μg/mL hygromycin and further confirmed by RT–PCR. The *Arabidopsis* actin2 (AT3G18780.2) gene was used as the internal control. We selected three independent T2 overexpression lines to evaluate the oil content and fatty acid composition in transgenic *Arabidopsis* seeds. Using C17:0 FA as an internal standard, the oil content and fatty acid composition were quantified using gas chromatography-mass spectrometry (GC–MS).

## 5. Conclusions

The genome-wide identification and comprehensive analysis of nsLTPs revealed the number of nsLTPs varied among the sesame varieties, and there were 52 *SiLTPs* in the reference genome sequences of “Zhongzhi13”. Types I and XI were the most abundant nsLTPs in sesame. The *SiLTPs* are expressed in various sesame plant tissues. They may play important roles during developmental and reproduction processes in sesame by interacting with various transcription factors. *SiLTPI.23* and *SiLTPI.28* were identified as the candidate genes associated with high oil content in sesame. Overexpression of *SiLTPI.23* in *Arabidopsis* promoted the oil content and regulated the fatty acids composition in transgenic *Arabidopsis* seeds, confirming the presumed function of this candidate gene. Overall, this study offered valuable information for the functional characterization of *SiLTPs* and for genetic improvement of oil content in sesame.

## Data Availability

Gene sequence information of nsLTPs in sesame is available at the *Sesamum indicum* genome database (Sinbase 2.0, http://www.sesame-bioinfo.org/Sinbase2.0).

## Supplementary Materials

The following are available online at https://www.mdpi.com/article/10.3390/ijms22105291/s1, Figure S1: The phylogenetic tree of all the nsLTP proteins of five sesame varieties. Different color of arcs represents different types of nsLTPs. The different color of lines represents different varieties. Yellow lines represent Baizhima, green lines represent Mishuozhima, purple lines represent Swetha, blue lines represent Yuzhi11, and red lines represent Zhognzhi13. The black dot represents the support values of the phylogenetic trees. Figure S2: The expressed genes in HO and LO sesame seeds in different periods. Table S1: Putative *SiLTPs* identified in the genome of sesame varieties. (a) ECM, the eight-cysteine motif; (b) AA, the number of amino acids; (c) SP, signal peptide; (d) MP, mature protein. Table S2: The nsLTP genes in *Arabidopsis thaliana*, *Brassica rapa* and *Oryza sativa*. Table S3: List of primers used for quantitative real-time RT–PCR analysis. Table S4: List of primers used for PCR analysis.
