# Supplementary figures and images for: Genome-Wide Analysis of nsLTP Gene Family and Identification of SiLTPs Contributing to High Oil Accumulation in Sesame (Sesamum indicum L.)
Source: Int J Mol Sci. 2021 May 18;22(10):5291. doi: 10.3390/ijms22105291 (PMC8157352; doi:10.3390/ijms22105291)

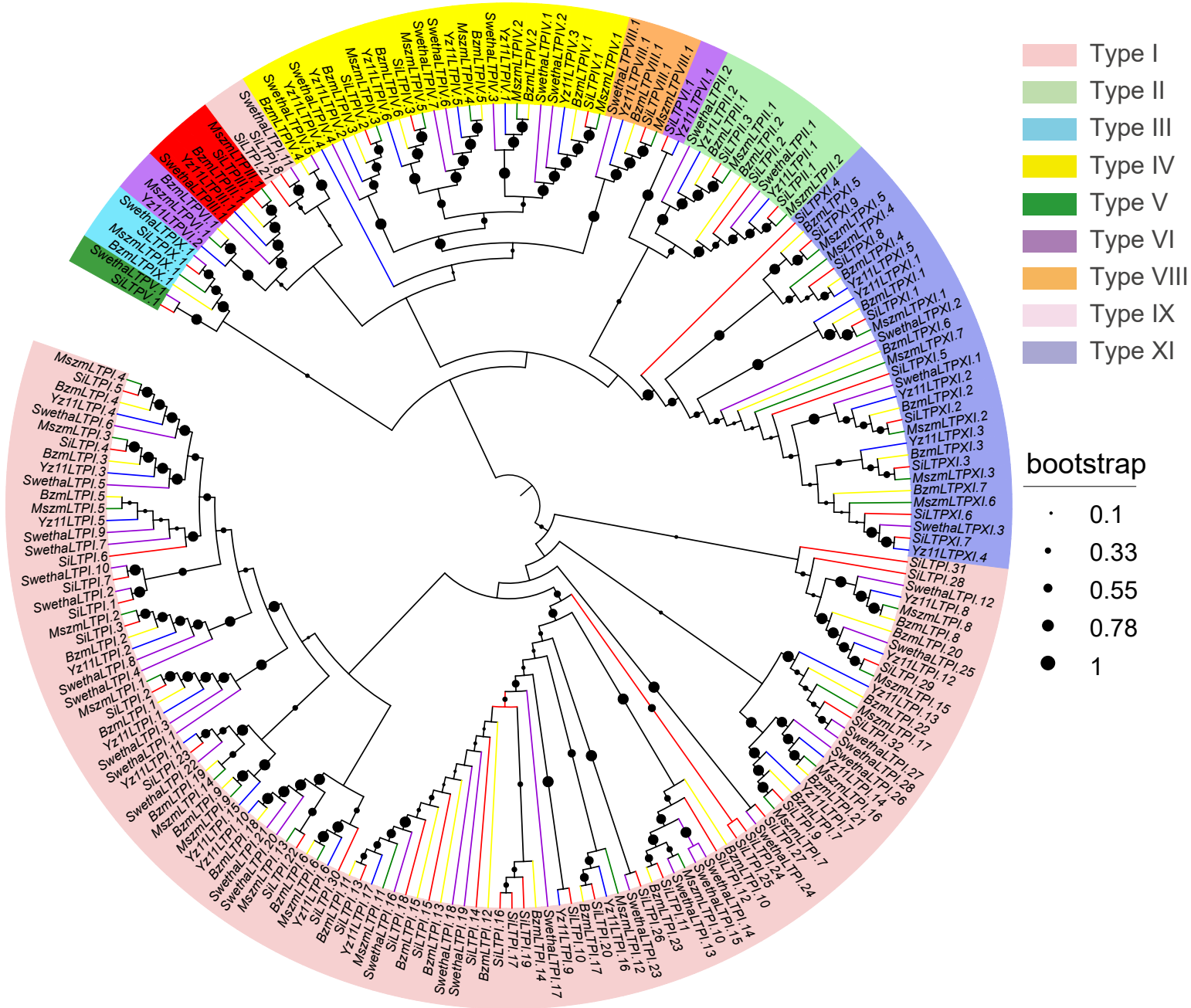

Supplement: Supplementary file 1 [file ijms-22-05291-s001.zip › ijms-1168850-supplementary/Figure S1.pdf]

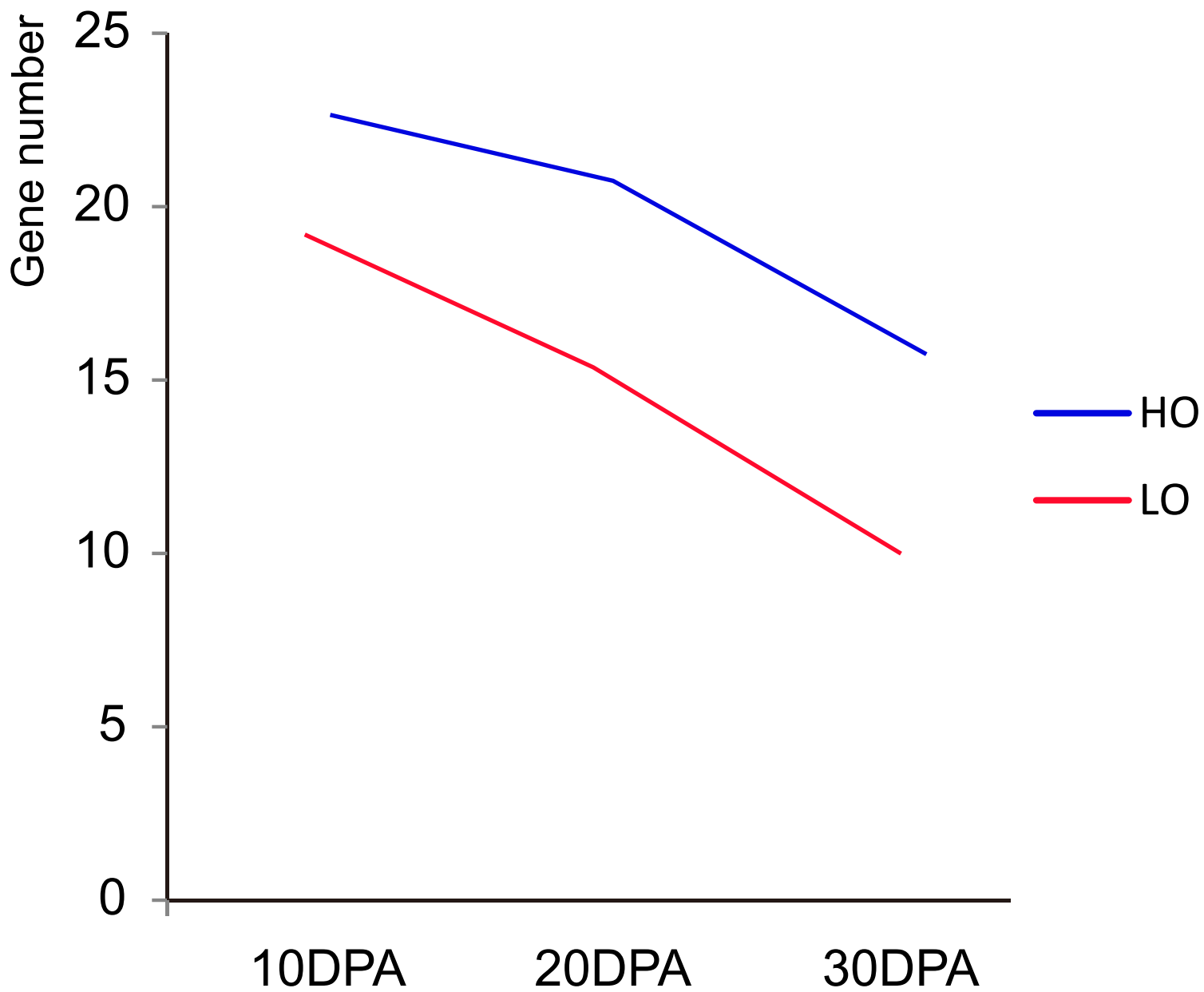

Supplement: Supplementary file 1 [file ijms-22-05291-s001.zip › ijms-1168850-supplementary/Figure S2.pdf]
